# A Novel Microtubule-Binding Drug Attenuates and Reverses Protein Aggregation in Animal Models of Alzheimer’s Disease

**DOI:** 10.3389/fnmol.2019.00310

**Published:** 2019-12-12

**Authors:** Samuel Kakraba, Srinivas Ayyadevara, Narsimha Reddy Penthala, Meenakshisundaram Balasubramaniam, Akshatha Ganne, Ling Liu, Ramani Alla, Shoban Babu Bommagani, Steven W. Barger, W. Sue T. Griffin, Peter A. Crooks, Robert J. Shmookler Reis

**Affiliations:** ^1^BioInformatics Program, University of Arkansas for Medical Sciences and University of Arkansas at Little Rock, Little Rock, AR, United States; ^2^Central Arkansas Veterans Healthcare Service, Little Rock, AR, United States; ^3^Department of Geriatrics, University of Arkansas for Medical Sciences, Little Rock, AR, United States; ^4^Department of Pharmaceutical Sciences, University of Arkansas for Medical Sciences, Little Rock, AR, United States

**Keywords:** Alzheimer’s disease, protein aggregation, GFAP (Glial Fibrillary Acidic Protein), tubulin, combretastatin, anti-aggregant activity

## Abstract

Age-progressive neurodegenerative pathologies, including Alzheimer’s disease (AD), are distinguished and diagnosed by disease-specific components of intra- or extra-cellular aggregates. Increasing evidence suggests that neuroinflammation promotes protein aggregation, and is involved in the etiology of neurological diseases. We synthesized and tested analogs of the naturally occurring tubulin-binding compound, combretastatin A-4. One such analog, PNR502, markedly reduced the quantity of Alzheimer-associated amyloid aggregates in the BRI-Aβ_1–42_ mouse model of AD, while blunting the ability of the pro-inflammatory cytokine IL-1β to raise levels of amyloid plaque and its protein precursors in a neuronal cell-culture model. In transgenic *Caenorhabditis elegans* (*C. elegans*) strains that express human Aβ_1–42_ in muscle or neurons, PNR502 rescued Aβ-induced disruption of motility (3.8-fold, *P* < 0.0001) or chemotaxis (1.8-fold, *P* < 0.05), respectively. Moreover, in *C. elegans* with neuronal expression of Aβ_1–42_, a single day of PNR502 exposure reverses the chemotaxis deficit by 54% (*P* < 0.01), actually exceeding the protection from longer exposure. Moreover, continuous PNR502 treatment extends nematode lifespan 23% (*P* ≤ 0.001). Given that PNR502 can slow, prevent, or reverse Alzheimer-like protein aggregation in human-cell-culture and animal models, and that its principal predicted and observed binding targets are proteins previously implicated in Alzheimer’s, we propose that PNR502 has therapeutic potential to inhibit cerebral Aβ_1–42_ aggregation and prevent or reverse neurodegeneration.

## Introduction

Aging affects every organ in the body and is an important risk factor for many progressive diseases including most neurodegenerative disorders (David et al., 2010; Niccoli and Partridge, 2012). With the rise in the average age of our population, there has been a disproportionate increase in neurological pathologies such as Alzheimer’s disease (AD), Parkinson’s disease (PD), and Huntington’s disease (HD; Calabrese et al., 2010; Morawe et al., 2012). AD is characterized by accumulation in the brain of intracellular neurofibrillary tangles (aggregates that contain hyperphosphorylated tau protein) and extracellular amyloid plaque (largely comprising Aβ_1–42_ peptide; Masters et al., 2015). Neocortex and hippocampus, regions of the brain associated with cognition and memory, are most severely impacted by AD (Frisoni et al., 1999). Genetic factors such as ApoE polymorphism, and non-genetic factors including diet, have been implicated in the risk level and etiology of AD (Ferri et al., 2005; Rizzi et al., 2014; Parcon et al., 2018).

Many deteriorative changes that accompany normal aging have been attributed to chronic inflammation, which is also strongly implicated in the development of AD (Aisen, 1996; Akiyama et al., 2000; De Felice and Ferreira, 2014). A variety of nonsteroidal anti-inflammatory drugs (NSAIDs) have been demonstrated to reduce the incidence of AD and other neurodegenerative disorders in prospective trials and in model organisms (Vlad et al., 2008; Varvel et al., 2009; Tasaki et al., 2012; Zhang et al., 2018). The same studies also found NSAIDs protect against cardiovascular and cerebrovascular disease (Patrono, 2013; Maestrini et al., 2018) and several types of adult-onset cancer (Di Francesco et al., 2015). Other, non-pharmacological strategies commonly employed to reduce the risk of AD include supplementation of polyunsaturated, ω-3 fatty acids (Lim et al., 2005), physical activity (Roach et al., 2011), and cognitive engagement (Maci et al., 2012). None of these strategies, however, and no drugs currently on the market or in clinical trials, have been shown to be effective in reversal of AD dementia once it is diagnosed, nor in slowing progression of disease. Drugs that have been studied as potential therapeutic agents for AD include acetylcholinesterase inhibitors (Speck-Planche et al., 2012), monoamine oxidase inhibitors, antioxidants, N-methyl-D-aspartate antagonists (NMDA), and anti-inflammatory drugs (Marlatt et al., 2005; Wong, 2005; Daniels et al., 2016).

Combretastatin A4 [*cis*-1-(3,4,5-trimethoxyphenyl)-2-(3-hydroxy-4-methoxyphenyl) ethane; CA4] has shown promise as an anti-cancer agent selectively toxic against diverse cancer cell lines. It is reported to inhibit cell growth even at 7 nM and to inhibit tubulin polymerization by 50% at much higher concentrations (IC_50_ = 2.5 mM; Lin et al., 1988). Combretastatin A4 was isolated from the *Cambretum caffrum* tree used in traditional medicine, and is the most potent of several compounds isolated from this plant with respect to anti-mitotic and cytotoxic activity, and for inhibition of tubulin polymerization (Lin et al., 1988; Pettit et al., 1989). A water-soluble version of Combretastatin A4, its disodium phosphate (CA4P), has been tested in clinical trials as a cancer chemotherapeutic agent (West and Price, 2004; Meyer et al., 2009).

In the present study, we show that a combretastatin analog, PNR502, can prevent and even reverse AD-like protein aggregation and associated functional/behavioral declines in *Caenorhabditis elegans* (*C. elegans*) models of Aβ_1–42_-induced amyloid deposition. It also extends nematode survival, either unstressed or during acute oxidative stress, and reduces cerebral aggregation in a mouse model of AD-like amyloidosis. We synthesized biotinyl derivatives of PNR502, which we used to isolate and identify its principal binding targets. PNR502 binds tubulin, as reported previously for combretastatin A4 (Pettit et al., 1989), and it binds even more avidly to Glial Fibrillary Acidic Protein (GFAP), an intermediate-filament protein most notably expressed in astrocytes and supporting their interactions with neurons (Figueiredo et al., 2008; Suzumura, 2013; Tsuda and Inoue, 2016).

## Materials and Methods

### *C. elegans* Strains

All nematode strains used in this study were obtained from the Caenorhabditis Genetics Center (CGC; Minneapolis, MN, USA). They comprise wild-type Bristol-N2 [DRM stock]; CL4176 [*smg-1*^ts^; *myo-3p*::*Aβ*_1–42_::*let-851* 3′-UTR; *rol-6(su1006)*] expressing human Aβ_1–42_ in muscle; and CL2355 [*smg-1*^ts^; *snb-1::Aβ*_1–42_::3′-UTR(long)*; mtl-2::gfp*] expressing human Aβ_1–42_ in all neurons. Strains were maintained at 20°C on 2% (w/v) agar plates containing nematode growth medium (NGM), overlaid with *E. coli* strain OP50 unless otherwise noted.

### Paralysis and Chemotaxis Assays in Aβ-Transgenic *Nematode* Strains CL4176 and CL2355

Transgenic *C. elegans* strains, capable of induction to express Aβ_1–42_ in muscle (CL4176) or in neurons (CL2355), were maintained at 20°C with ample *E. coli* (OP50) bacteria, and lysed at day 3.5 post-hatch (adult day 1), releasing unlaid eggs to generate a synchronized cohort. Eggs were plated on 100-mm Petri dishes containing NGM-agar seeded in a central area with OP50 bacteria plus PNR502 or vehicle (to a final concentration of 0.02% v/v DMSO). Worms were either upshifted to 25.5°C at the L3-L4 transition to induce expression of the human Aβ_1–42_ transgene and assayed after a further 48 h, or were aged without induction and assayed at a series of later times. Paralysis (Dostal and Link, 2010) and chemotaxis (Dosanjh et al., 2010) assays were performed as described previously (Ayyadevara et al., 2016b).

### Pulldown of PNR502 Binding Targets

AD hippocampal tissue was flash frozen and stored at −80°C, and then pulverized in a mortar and pestle cooled on dry ice, just prior to isolation of total protein as described previously (Ayyadevara et al., 2016b). Equal protein contents were pooled from three tissue lysates and incubated with biotinyl-PNR502 (10 μM) for 5 h. Retained protein was digested with 5 μg/ml trypsin (Promega) for 2 h at 37°C. Bound, lightly digested protein was recovered on streptavidin-coated magnetic beads (Thermo Fisher Scientific, Waltham, MA, USA), and eluted peptides were analyzed by LC-MS/MS as described (Ayyadevara et al., 2016b,d).

### RNA Interference

Selected genes, encoding abundant proteins identified from PNR502 pulldown, were subjected to RNAi knockdown by feeding worms on HT115 bacterial sublines from the Ahringer library (Kamath et al., 2003). Synchronously harvested eggs were transferred to plates seeded with *E. coli* HT115 (DE3) bacteria that transcribe double-stranded RNA corresponding to an exonic segment of the targeted gene, cloned into the L4440 plasmid multiple-cloning site (Kamath et al., 2003). Control worms were fed bacteria carrying L4440 without an exonic insert (“feeding vector” or FV controls).

### Lifespan Studies

Worms were lysed to collect synchronized eggs, which were plated on control plates containing varying concentrations of PNR502, or DMSO vehicle alone (for a final concentration of 0.02% v/v DMSO). Survival worms were picked at the L4 larval stage, and transferred to fresh plates daily for 7 days, then on alternate days, scoring worms as alive if they moved spontaneously or in response to gentle prodding (Bharill et al., 2013; Ayyadevara et al., 2016a). Worms lost for reasons other than natural death were censored from mortality calculations.

### Effect of PNR502 on Protein Aggregation in Human Cells

Neuronal (SH-SY5Y-APP_Sw_) and glial (T98G) cells were grown as previously described (Liu et al., 2005). SY5Y-APP_Sw_ cells expressing an aggregation-prone double mutant of amyloid precursor protein (APP_Sw_) were grown in DMEM plus 10% (v/v) fetal bovine serum (FBS) at 37°C. Cells were suspended in trypsin/EDTA and rinsed in buffer prior to replating or harvesting. Prior to assay, cells were grown for 48 h in the presence of 5-μM PNR502 dissolved in DMSO (0.02% final concentration) or the same amount of DMSO solvent for control cells. T98G glial cells were plated in 35-mm dishes at 500,000 cells/dish, as above, and incubated 48 h at 37°C prior to treatment with 30-nM soluble amyloid precursor protein α (sAPPα) for 16 h. To assess protective effects of PNR502, cells were treated with 10-nM PNR502, either simultaneous with sAPPα or beginning 1 h prior to sAPPα addition (pre-treatment). Cells were harvested, total protein was isolated, and aggregate proteins were purified as described below.

### Western-Blotting Analysis of NT2 Cells for p38/MAPK and ApoE

NT2 cells (the NTera2 human embryonal carcinoma cell line, American Type Culture Collection, Manassas, VA, USA) were maintained in Dulbecco’s modified Eagle medium (DMEM; Invitrogen/Life Technologies, Grand Island, NY, USA), supplemented to 10% v/v with FBS. Cells were exposed to 30-ng/ml IL-1β, or to IL-1β plus 10-nM PNR502, for 24 h. The cells were harvested and their proteins extracted in lysis buffer (50-mM Tris-HCl, pH 7.5, 150-mM NaCl, 1% w/v Nonidet P40, 0.1% SDS, 0.5% sodium deoxycholate) and quantified with Bradford reagent (Bio-Rad). Protein aliquots (100 μg) were electrophoresed 2 h at 100 V on a 4%–20% gradient bis-tris acrylamide gel (BioRad Life Science, Hercules CA, USA), and transferred to nitrocellulose membranes. Blots, after pre-incubation with BSA blocker (Pierce), were probed with rabbit antibody to active human p38/MAPK (Cell Signaling, 1:100 dilution) or goat anti-human ApoE (Calbiochem) overnight at 4°C. After washes, the membrane was incubated with HRP-conjugated goat anti-rabbit secondary antibody (AbCam, 1:10,000 dilution) or rabbit anti-goat IgG (Rockland Immunochemicals, Gilbertsville, PA, USA) for 1-h at room temperature. After washes, the membrane was developed with ECL chemiluminescence detection kit (Pierce). Data were digitized and analyzed using ImageJ software (NIH).

### Thioflavin-T and Antibody Staining of Amyloid in SY5Y-APP_Sw_ Cells

Cells were replated and grown (typically 48 h following drug exposure or introduction of small interfering RNA (siRNA; Sigma/Millipore), in RNAmax lipofection reagent (Thermo Fisher Scientific, Waltham, MA, USA), fixed in formaldehyde (4% v/v) and stained in a dark container with 0.1% w/v Thioflavin T. After four washes in PBS, cells were covered with Antifade + DAPI (Life Technologies, Grand Island, NY, USA) and fluorescence was captured using appropriate filters (DAPI/blue and Thioflavin T/green) with a Nikon DS-Fi2 camera mounted on a Nikon C2 inverted microscope with motorized stage for automated well-by-well imaging. Immunohistochemistry methods were described previously (Balasubramaniam et al., 2018). Briefly, fixed cells were probed with mouse antibody to human Aβ [ab 11132 (AbCam), 1:400 dilution] for 2 h. After washes in PBS, cells were incubated 30 min at 22°C with goat anti-mouse secondary antibody coupled to Alexa488 (Life Technologies, 1:500 dilution) and imaged with a Keyence fluorescence microscope.

### Isolation of Aggregate Proteins

*C. elegans* adults, cultured human neuronal cells, or mouse cerebra were collected, rinsed, drained of liquid, and flash-frozen in liquid nitrogen, pulverized in a dry-ice-cooled mortar, and suspended in buffer with nonionic detergent (20-mM Hepes buffer, pH 7.4, 0.3-M NaCl, 2-mM MgCl_2_, 1% NP40, and protease/phosphatase inhibitors [CalBiochem]), all at 0°C (Ayyadevara et al., 2015). Lysate was centrifuged (5 min, 3,000 rpm at 4°C) to remove debris. After removal of cytosolic proteins (soluble in 1% NP40 nonionic detergent) as the centrifugation supernatant (18 min, 13,000× *g* at 4°C), protein pellets including aggregates were brought to pH 7.4 with 0.1 M HEPES buffer containing ionic detergent (at final concentrations of 1% v/v sarcosyl, 5-mM EDTA), and centrifuged 30 min at 100,000× *g*. The pellet and supernatant are the detergent-insoluble and -soluble fractions, respectively (Ayyadevara et al., 2015). The sarcosyl-insoluble pellet was resuspended in Laemmli loading buffer (containing 50-mM freshly diluted dithiothreitol, 2% v/v SDS), heated 2 min at 95°C to dissolve proteins, and electrophoresed on gels of 10% w/v polyacrylamide with 1% w/v SDS.

### Structural Modeling of Target Proteins

The structure of the tubulin β chain (PDBid: 1Z2B), determined by X-ray diffraction of crystals, was retrieved from the Protein Data Bank, PDB^1^. Because the full-length structure of GFAP had not been determined from X-ray or NMR data, we predicted its structure computationally with I-TASSER. This web server^2^ first identifies polypeptide segments for which structures have been ascertained and deposited in PDB (fold recognition), from which full-length structures are assembled. I-TASSER fills in indeterminate or unstructured regions by *ab-initio* modeling *via* replica-exchange (Monte Carlo simulations), identifies low free-energy states, and uses them as inputs for iterative assembly simulations, which are refined by the inclusion of steric constraints identified in the preceding steps. We then predicted the structure of the bimolecular complexes of GFAP and tubulin β chain using Hex^3^, a protein-protein docking algorithm. Finally, we modeled energy-minimized conformers of fully solvated GFAP, tubulin β chain, and their complex, using the GROMACS simulation package. This step is essential since cytosolic biomolecules are fully hydrated and balanced by counterions, whereas crystals are not.

### Protein-Ligand Docking of PNR502 to Target Proteins

Both GFAP and tubulin structures were converted to Autodock format. Primary docking studies were performed using AutoDock Vina^4^ with the Raccoon interface (Forli et al., 2016). To ensure unbiased blind docking, the target box was set to enclose the entire molecule, allowing the ligand to find its optimal binding pockets. The energy-minimized binding pose, selected from 10 replicate simulations, was used for further calculations. Docking poses and binding residues were viewed and analyzed with the BIOVIA Discovery Studio.

### Statistical Analyses

Significance of survival differences was ascertained by Gehan-Wilcoxon log-rank tests. For protein aggregation, chemotaxis, and paralysis, differences between control and experimental groups were assessed for significance by the Fisher-Behrens heteroscedastic *t*-test (appropriate to samples of unequal or unknown variance), treating each experiment as a single point. Two-tailed tests were employed when the direction of change was not known, but 1-tailed tests are appropriate once that direction has been firmly established. For culture assessments after staining with Thioflavin-T or antibody, means were taken for 8–9 fields per well, and means per well were treated as individual points comprising each treatment group. Within experiments, differences in proportions (fractional paralysis or chemotaxis) were evaluated by chi-squared or Fisher exact tests.

## Results

**Figure 1:**
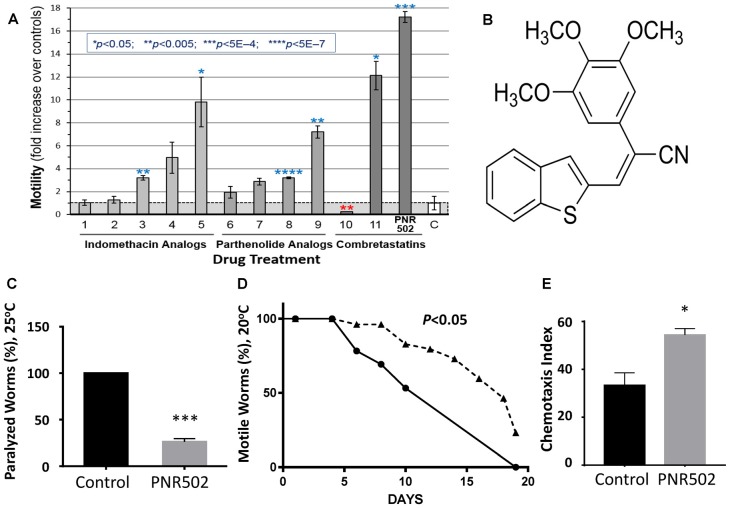
PNR502 is exceptionally effective in reducing amyloid toxicity in *Caenorhabditis elegans* (*C. elegans*). **(A)** Three families of drugs ameliorate a locomotion deficit in *C. elegans* (strain CL4176) expressing human Aβ_1–42_ in muscle. Worms were induced at the L4/adult transition, by upshift from 20° to 25.5°C, and then maintained for 41 h on solid media with the indicated compounds (at 10 μM) or vehicle. For motility assay, worms were placed 20 per well, in S basal medium in a flat-bottom 96-well plate. Movement was monitored with wMicrotracker (NemaMetrix), a motion-tracking device that records disruptions of 4 infrared beams per well. Error bars show standard deviations. Significance, in 2-tailed *t* tests, of difference between treated groups and controls (bar “C” at right): *P < 0.05; **P < 0.005; ***P < 5E-4; ****P < 5E-7. **(B)** The structure of PNR502. **(C)** The paralyzed fraction of CL4176 worms was reduced 75% by treatment with 10-μM PNR502 for 48 h after induction (as in A, assessed manually). **(D)** Uninduced CL4176 worms show progressive paralysis as they age at 20°C. The median time to paralysis is delayed 1.8-fold by continuous exposure to 10-μM PNR502. **(E)** CL2355 nematodes express human Aβ_1–42_ in all neurons, resulting in impaired chemotaxis 48 h after induction. Exposure to 5-μM PNR502 preserves chemotaxis relative to vehicle-only control worms. **(C,E)** Treated groups differ from controls by the Fisher exact test: *P < 0.01; ***P < 0.0001.

Accumulation of protein aggregates in and around cells is a hallmark of aging and many age-associated disorders (David et al., 2010; Ayyadevara et al., 2015, 2016a,b,c,d; Walther et al., 2015). Compounds that reduce protein aggregation have been shown to lower amyloid, tau and α-synuclein toxicity in human cells (Amijee et al., 2009; Rinderspacher et al., 2009; Bulic et al., 2010), and recently to extend *C. elegans* lifespan and healthspan (Ayyadevara et al., 2013, 2017; Cuanalo-Contreras et al., 2017). We synthesized analogs of several plant-derived drugs, in search of novel agents that delay or prevent aggregation-mediated diseases. We initially screened molecules from three drug libraries, in a *C. elegans* strain that expresses low levels of human Aβ_1–42_ in muscle, resulting in age-dependent deposition of amyloid plaque and a progressive loss of motility (Figure 1A). The most effective compound tested, enhancing worm motility 17-fold, was PNR502 (structure shown in Figure 1B), derived from the tubulin-binding drug combretastatin A4.

### PNR502 Rescues Motility and Chemotaxis Defects in *C. elegans* Models of Aβ Amyloidosis

Protein aggregation increases with age and is associated with neurodegeneration and other age-dependent diseases (Brehme et al., 2014; Ayyadevara et al., 2015, 2016b; Mukherjee et al., 2015). We assessed the effects of PNR502 on aggregation in *C. elegans* transgenic models expressing “seed” proteins characteristic of human neurodegenerative diseases. Two of the nematode models were designed to simulate amyloid plaque formation, characteristic of AD. Adult worms of strain CL4176 accrue amyloid plaque and become paralyzed ~2 days after induction of human Aβ_1–42_ in body-wall muscle, but paralysis is reduced more than 4-fold in worms exposed concurrently to 10-μM PNR502 (Figure 1C). We modified this model to simulate the progressive nature of AD, by monitoring uninduced worms as they slowly accumulate amyloid while aging at 20°C. Beginning at adult day 5, and progressing over the ensuing 2 weeks, motility declines in untreated worms, presumably due to leaky expression from the Aβ_1–42_ transgene (Ayyadevara et al., 2015); PNR502 treatment delays paralysis 1.8-fold (Figure 1D).

Strain CL2355 can be induced by temperature upshift (or by aging in the absence of upshift) to express Aβ_1–42_ in all neurons, which disrupts the normal chemotactic response (e.g., movement toward N-butanol) 2 days later (Dostal and Link, 2010). This behavioral trait is dramatically rescued in worms exposed to PNR502 (*P* < 0.05; Figure 1E).

### PNR502 Rescues Chemotaxis in a *C. elegans* Model of Age-Dependent Amyloid Neuropathy

**Figure 2:**
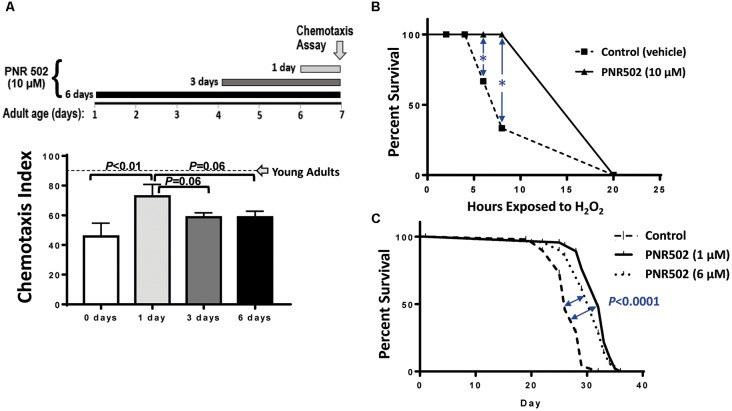
PNR502 reduces neurotoxicity, sensitivity to oxidative stress, and mortality in *C. elegans*. **(A)** *C. elegans* adult worms (strain CL2355, without induction), a model of Alzheimer’s disease (AD)-like amyloidosis, were treated with 10-μM PNR502 for 1, 3 or 6 days just prior to assaying chemotaxis towards n-butanol. Worms treated for 1 day differ from controls at *P* < 0.01 (histogram). Worms treated 3 or 6 days differ from controls at *P* < 0.05, and may also differ from the 1-day treatment group (*P* = 0.06). Error bars show standard deviations. The dashed line indicates normal chemotaxis of uninduced adults at day 1. **(B)** *C. elegans* wild-type worms (Bristol-N2 [DRM]) were exposed to 10-μM PNR502 or vehicle alone (controls) and monitored for survival during exposure to 5-mM hydrogen peroxide as an oxidative stress. *Significance of survival differences at 6 and 8 h, by two-sided *t*-test: *P* < 0.01. **(C)** Worms exposed to 1- or 6-μM PNR502, or vehicle alone, were monitored for survival at 20°C under benign conditions to assess lifespan. Significance of survival-curve differences, by Gehan-Wilcoxon log-rank tests: *P* < 0.05 for stress response (B, each *N* = 30), and *P* < 1E-4 for lifespan (C, each *N* = 40). Data displayed were combined from two independent experiments of each type **(B,C)**.

*C. elegans* strain CL2355 also exhibits an age-dependent loss of chemotaxis in the absence of transgene induction (Ayyadevara et al., 2015), just as their uninduced, muscle-expressing counterparts (CL4176) gradually became paralyzed with age (Figure 1D). We took advantage of this progressive phenotype to assess whether PNR502 can reverse aggregation-mediated symptoms. Adult worms were tested for chemotaxis to n-butanol on adult day 7, immediately preceded by 1, 3, or 6 days of exposure to 10-μM PNR502. Without drug intervention (“0 days”), 7-day-old adults showed only 47% chemotaxis (Figure 2A), far below the 90% index shown by day-1 adults (Ayyadevara et al., 2015). Just a single day of PNR502 treatment restored ~2/3 of the deficit relative to day-1 chemotaxis, which exceeded (although not significantly) the rescue observed after 3 or 6 days of PNR502 exposure (Figure 2A). Results were similar for age-dependent polyglutamine::YFP aggregation, in a *C. elegans* model of HD (strain AM141): rescue by 1-day PNR502 treatment was only slightly less effective than (but not significantly different from) exposure for 3 or 6 days (data not shown).

### PNR502 Improves Survival of Oxidative Stress and Extends Lifespan of Wild-Type Nematodes

Survival of oxidative stress (hydrogen peroxide or paraquat) is strongly and positively correlated with lifespan, among *C. elegans* groups aging normally or with lifespan extended by genetic or drug interventions (Ayyadevara et al., 2005, 2013, 2016a; Bharill et al., 2013). Drugs that inhibit protein aggregation also extend both mean and maximal lifespan (Ayyadevara et al., 2013; Cuanalo-Contreras et al., 2017). To assess the impact of PNR502 on oxidative-stress resistance, 10-μM PNR502 or vehicle alone was administered to wild-type *C. elegans* [the relatively long-lived N2_DRM_ stock of strain Bristol-N2 (Gems and Riddle, 2000)] from hatch to adult day 3, and their survival was then assessed in 5-mM H_2_O_2_ (oxidative stress; Figure 2B). After 6 and 8 h. of oxidative stress, 30 and 60% of control worms died, respectively, whereas none of the PNR502-treated worms had died (each *P* < 0.01).

We then assessed normal lifespan under unstressed conditions. Worms fed PNR502 at 1 or 6 μM continuously from the L4/adult transition had mean, median, and maximal (90% mortality) lifespans extended by 15–20% (Figure 2C; each *P* < 0.0001 by Gehan-Wilcoxon log-rank test), relative to vehicle-only controls. Very similar results (not shown) were obtained when drug treatments began at egg hatch rather than upon completion of larval development.

### PNR502 Reduces Protein Aggregation in Cultured Human Neuronal and Glial Cells

Protein aggregation was monitored in human neuronal and glial cell lines *in vitro*, following mock treatment (vehicle only) or treatment with PNR502. Cell lines T98G (human glioblastoma cells) and SY5Y-APP_Sw_ (human neuroblastoma) were each cultured 26 h at 37°C, allowing ~1 population doubling, in DMEM medium containing 10% fetal calf serum, with or without PNR502 at subtoxic drug concentrations for each cell type (i.e., 10 nM for T98G; 5 μM for SY5Y-APP_Sw_).

**Figure 3:**
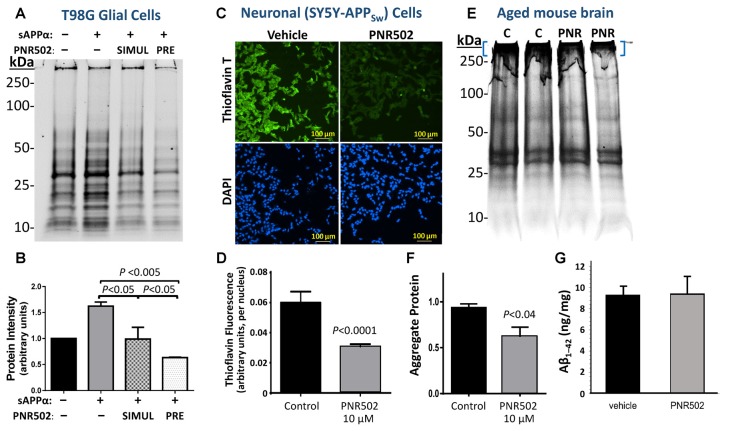
PNR502 reduces protein aggregation in human glial and neuronal cells in culture, and in mouse cerebra *in vivo*. **(A,B)** T98G human glial cells were cultured 26 h either untreated or in the presence of 30-nM sAPPα. The right two lanes are from cells with either prior or simultaneous addition of 10-nM PNR502. Untreated cells received vehicle alone (controls). Insoluble aggregates were then isolated and their full-lane protein content was quantified by SYPRO-Ruby staining (Thermo Fisher Scientific, Waltham, MA, USA) of electrophoresed proteins (*N* = 5 per group). Error bars show standard deviations; *P*-values are from 2-tailed heteroscedastic *t*-tests. **(C,D)** SY5Y-APP_Sw_ human neuroblastoma cells were cultured for 2 days in the presence or absence of 5-μM PNR502, after which amyloid was stained with Thioflavin T and quantified from fluorescence images (see “Materials and Methods” section). Significance of differences from controls was determined by 2-tailed heteroscedastic *t-*tests. **(E,F)** Sarcosyl-insoluble aggregates were isolated from cerebra of adult (12-month-old) mice that had been injected I.P. with vehicle or PNR502 (5.7 μM/kg/day) for 10 successive days. Aggregates were resuspended in Laemmli buffer (2% SDS, 50-mM β-mecaptoethanol) at 95°C electrophoresed in SDS/polyacrylamide gels, and stained with SYPRO Ruby. **(F)** Protein staining is summarized for sarcosyl-insoluble aggregates isolated from mouse cerebra (*N* = 5 per group). Only protein complexes of very low mobility (blue brackets in **E**) were considered in this quantitation, but the reduction in intensity after PNR502 was similar for gel regions of higher mobility, and for full-lane scans (not shown). **(G)** Total Aβ_1–42_ in mouse cerebra, quantified with primary and fluor-tagged secondary antibodies, was not affected by PNR502 injections.

T98G glial cells were induced to accumulate aggregates by exposure to 30-nM soluble APPα (sAPPα), a pro-inflammatory treatment, concurrent with or preceding PNR502 treatment (*N* = 3 per group). Aggregates were isolated as sarcosyl-insoluble material, resuspended by heating to >95°C in Laemmli buffer, electrophoresed on polyacrylamide-SDS gels, and then quantified by SYPRO Ruby staining (Figure 3A) as described in the “Materials and Methods” section. Results (areas under full lane-scan profiles) are summarized in Figure 3B, with each sAPPα-exposed value normalized to simultaneous untreated control cells (left lane and bar). Addition of sAPPα alone increased insoluble aggregates by 64%, but this response was entirely blocked by simultaneous exposure to 10-nM PNR502 (compare “SIMUL” PNR502 to sAPPα alone). When PNR502 was added 1 h prior to sAPPα (“PRE” PNR502), insoluble aggregates were reduced by a further 38% relative to untreated or simultaneously-treated cells (*P* < 0.05 for the latter comparison, by 2-tailed *t*-test), or by 60% relative to cells receiving sAPPα alone (*P* < 0.005), consistent with PNR502 not only impeding aggregate formation but also removing previously-formed aggregates from cultured human cells, just as observed in a *C. elegans* model of AD-like amyloid deposition (Figure 2A).

SY5Y-APP_Sw_ neuroblastoma cells were assessed for amyloid by staining with Thioflavin T (0.1% w/v), counterstaining nuclei with DAPI, and calculating the mean Thioflavin-T/amyloid fluorescence per nucleus over multiple fields (Ayyadevara et al., 2015). Typical images of amyloid fluorescence in PNR502- or vehicle-treated SY5Y-APP_Sw_ neuroblastoma cells are shown in Figure 3C. As summarized in Figure 3D, PNR502 reduced total fluorescence per cell by ~50% (*P* < 0.0001 by 2-tailed *t*-test).

**Figure 4:**
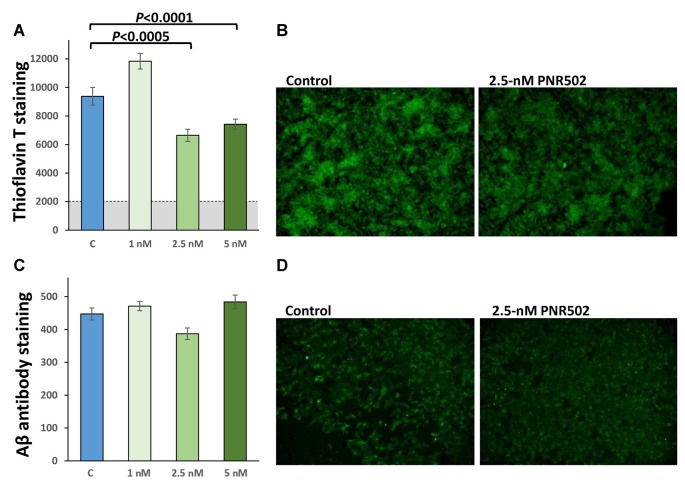
PNR502 reduces amyloid aggregation and shifts Aβ signal from punctate foci to diffuse background. SY5Y-APP_Sw_ cells were treated 48 h with the indicated concentrations of PNR502, and then fixed and stained in parallel with Thioflavin-T (total amyloid, **A,B**) or with antibody specific for Aβ_1–17_ (AbCam, ab11132; **C,D**). **(A,C)** Quantitations compared to six independent cultures per group, with *P*-values obtained from single-tailed *t*-tests appropriate when the direction of change is known.

To assess the effect of PNR502 specifically on Aβ, cultures were grown 48 h in the presence of PNR502, at concentrations that elicited no apparent toxic effects (1, 2.5, and 5 nM), or vehicle alone. Cells were then fixed and stained as described (Balasubramaniam et al., 2018), in parallel with Thioflavin T or antibody to Aβ_1–42_ (Figure 4). PNR502 treatment of SY5Y-APP_Sw_ cells suppressed punctate immunostaining with antibody to Aβ peptide, but this was more difficult to quantify because such antibodies also detect diffuse (unaggregated) Aβ_1–42_ which replaces the punctate signal (see Figure 4D). We thus rely on quantitation of cell staining by Thioflavin T (Figures 4A,B), which is specific to punctate aggregate foci such as β amyloid.

### PNR502 Reduces Cerebral Protein Aggregation in a Mouse Model of AD Amyloidosis

BRI-Aβ_1–42_ transgenic mice express the Aβ_1–42_ peptide conjoined by a furin-cleavable linkage to the BRI transmembrane protein. These mice develop amyloid plaque and a significant learning deficit prior to 1 year of age, attributed to excess Aβ_1–42_ release without APP overexpression (Lewis et al., 2001). BRI-Aβ_1–42_ mice at 12 months of age were injected intraperitoneally for 10 successive days with PNR502 (5.7 μM/kg/day) or with vehicle alone, to determine whether cerebral aggregation was altered or reversed by drug exposure. After euthanasia, sarcosyl-insoluble aggregates were isolated from individual mouse cerebra, electrophoresed on polyacrylamide gels (Figure 3E), and quantified by SYPRO Ruby staining (Thermo Fisher Scientific, Waltham, MA, USA) as above. PNR502 treatment reduced the total insoluble-protein signal by 18% (Figure 3E; quantitation not shown), while aggregates unable to enter the gel declined by >35% relative to controls (Figure 3F; adjusted *P* < 0.04 by 2-tailed *t*-test, *N* = 5 per group). However, the total quantity of Aβ_1–42_ in cerebral tissue (Figure 3G) was unchanged by PNR502 treatment.

### PNR502 Blocks Induction of Active p38/MAPK and ApoE by IL-1β

**Figure 5:**
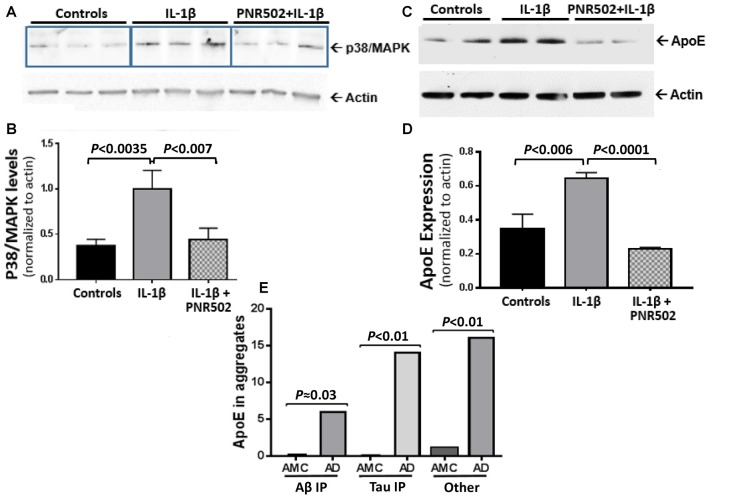
PNR502 blocks IL-1β-mediated increases in p38/MAPK and ApoE expression. **(A,B**) Primary neuronal cells, treated with IL-1β (30 ng/ml) *in vitro*, showed increased expression of p38/MAPK (quantified with antibody to the active form of p38/MAPK), but this response was blocked by pretreatment with 10-nM PNR502. The results are summarized in **(B)**, showing means ± SD from three independent experiments, each comprising triplicate wells (as in **A**) and combined for statistical analysis by 2-tailed heteroscedastic *t-*test. **(C)** ApoE is induced by IL-1β treatment of NT2 cells (16 h, 30 ng/ml), but blocked if they are concurrently exposed to 10-nM PNR502; results, summarized in **(D)**, show means ± SD from three independent experiments, each performed in duplicate. **(E)** Aβ_1–42_- and tau-containing aggregates were isolated by immuno-pulldown (IP) as described previously (Ayyadevara et al., 2016b). These two aggregate types, and total aggregates (without IP) were resuspended in buffer containing 1% (w/v) sarcosyl, pelleted by high-speed centrifugation, recovered and analyzed by proteomics as described (Ayyadevara et al., 2016b). ApoE was roughly quantified in hippocampal aggregates from AD or controls (AMC), based on spectral hits (mean ± SEM) for four samples per group.

Il-1β is a central mediator of neuroinflammation, which triggers the activation of p38/MAPK signaling (Sheng et al., 2001) that mediates the cellular response to cytokines and external stresses. Concurrent exposure to PNR502 blocks the IL-1β-initiated induction of active p38/MAPK in primary neurons (Figures 5A,B). NT2 (NTERA-2) human embryonal carcinoma cells respond to IL-1β with increased cellular levels of Apolipoprotein E (ApoE), which appears in detergent-insoluble aggregates from several tissues and promotes aggregation (Ayyadevara et al., 2016b,c,d). Pretreatment of neuronally differentiating NT2 cells with 10-nM PNR502 reverses the IL-1β-induced increase of ApoE expression (Figures 5C,D). ApoE abundance is markedly elevated in AD-derived hippocampus, as reported previously (Ayyadevara et al., 2016b) and as illustrated by proteomic quantitations summarized (Figure 5E) for three classes of hippocampal aggregates isolated from AD vs. age-matched controls (AMC).

### PNR502 Binds to Protein Aggregates From *C. elegans* Muscle and Human Brain

**Figure 6:**
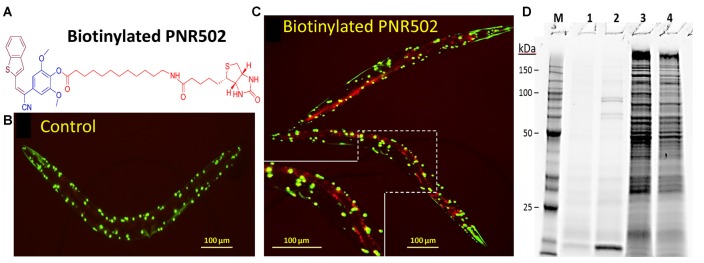
Biotinylated PNR502 localizes to aggregates in AM141 worms and is used to recover drug-adherent proteins. **(A)** Structure of biotinyl-PNR502. **(B,C)** AM141 adult worms were either untreated **(B)** or treated 26 h **(C)** with 10-μM PNR502 and then fed Alexa594-conjugated streptavidin (Thermo Fisher Scientific, Waltham, MA, USA). Fluorescence images were captured with a Nikon DS-Fi2 camera mounted on a Nikon C2 inverted microscope. Q40::YFP is displayed as green, and Alexa594 as red fluorescence. Yellow fluorescence in **(C)** indicates the superposition of Q40::YFP with PNR502-Alexa594. **(D)** Caudal hippocampi, from normal age-matched controls (AMC) or AD patients (pools of three per group), were lysed and incubated 2 h at 4°C with 5-μM PNR502 or biotinyl-PNR502. **M**, size markers; lanes 1–4, proteins recovered from: **1**, unmodified PNR502; **2**, biotinyl-PNR502; **3**, equivalent portion of flow-through for unmodified PNR502; **4**, flow-through from biotinyl-PNR502.

We synthesized two biotinylated versions of PNR502, one of which (the structure shown in Figure 6A) opposes aggregation as effectively as unmodified PNR502. Aggregates were assessed in *C. elegans* strain AM141, which expresses a polyglutamine reporter (Q40::YFP) in muscle, and thus serves as a model of glutamine-tract aggregation observed in HD and diverse ataxias (Morley et al., 2002). AM141 young adults were exposed to 10-μM biotinyl-PNR502 for 24 h, and then fed streptavidin coupled to Alexa594^TM^ fluor for 4 h. Controls never exposed to PNR502 contain only Q40::YFP foci in the body-wall muscle (Figure 6B; YFP is displayed as green). Alexa594-tagged biotinyl-PNR502 co-localizes with green Q40::YFP aggregates in muscle, resulting in yellow (red + green) foci that indicate PNR502 situated within aggregates, in addition to red fluorescence in gut cells, attributed to ingestion of Alexa-tagged streptavidin (Figure 6C).

Biotinylated PNR502 was incubated with fresh lysate from AD hippocampus (a pool from three individuals) and captured on streptavidin-coated magnetic beads. After stringent washes, proteins eluted from the beads were resuspended in Laemmli buffer at >95°C, and resolved by SDS-polyacrylamide gel electrophoresis. This protein-capture strategy reduced the complexity of proteins >30-fold [Figure 6D, compare lane 2 (capture) to lane 4 (flow-through)], implying high selectivity.

**Figure 7:**
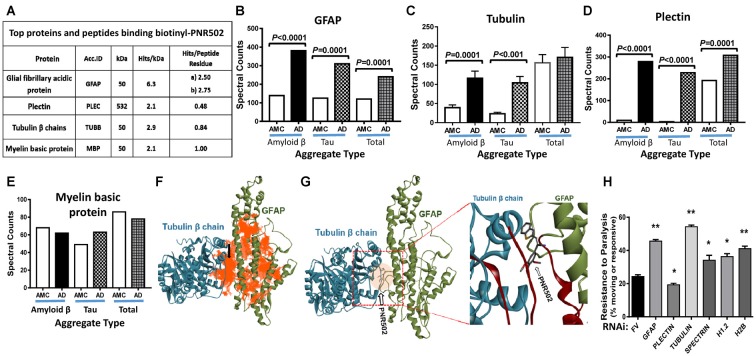
Three of the top four PNR502-binding proteins (based on spectral hits per peptide residue) are much more abundant in AD cortex than in controls and may play functional roles in aggregation. **(A)** PNR502 binds to GFAP, tubulin, plectin, and myelin basic protein (MBP). **(B–E)** These proteins were enriched in AD relative to AMC (control) aggregates isolated after IP with antibody to amyloid β or tau; more modest enrichments were also observed in the “total aggregate” fraction **(B,D)**. **(F)** Ribbon structures are shown for tubulin β chain in a complex with GFAP. Orange highlighting indicates regions of predicted PNR502 binding, chiefly in GFAP. **(G)** The preferred (lowest G) binding site of PNR502 lies at the interface between GFAP and β-tubulin; in the magnified view (right), the peptides identified by PNR502 pulldown are colored crimson. **(H)** RNAi knockdowns targeting GFAP, β-tubulin and histones H1.2 and H2B, significantly increase the unparalyzed fraction of CL4176 worms (those moving spontaneously or in response to gentle prodding), 48 h after Aβ_1–42_ synthesis was induced by upshift from 20°C to 25.5°C at the L3/L4 transition. Significance of differences between RNAi knockdowns and FV controls, by 2-tailed heteroscedastic *t*-tests: *P < 0.05; **P < 0.005. Error bars indicate standard deviations.

Lysate from Alzheimer’s caudal hippocampus (a pool from three subjects) was again incubated with biotinyl-PNR502 and partially digested with trypsin. Bound peptides were captured on streptavidin-coated beads, then washed and eluted for analysis by mass spectrometry (LC-MS/MS). Mild tryptic digestion was used to disperse aggregates while preserving sufficient local protein structure to maintain drug binding. Several highly-abundant proteins were identified (Figure 7A). GFAP is the most compelling candidate target of PNR502; two GFAP peptides had 2.2 and 2.4 times as many spectral hits per peptide residue as histone H2B, and even larger abundance ratios relative to other bound proteins (Figure 7A). Plectin is another candidate PNR502-binding protein that could mediate anti-aggregative activity. Additional PNR502-bound proteins with relatively high normalized abundance include tubulin β chain (TUBB), myelin basic protein (MBP), and Histone H2B (H2B3B). In two independent isolations, total spectral hits for GFAP were 2–2.5× as abundant as tubulin α- and β-chain hits combined (data not shown), despite similar molecular weights of GFAP (55 kDa) and tubulin chains (50 kDa). GFAP, tubulin and plectin were significantly enriched in aggregates from AD relative to AMC, isolated by immuno-pulldown (IP) with antibodies to Aβ_1–42_ (amyloid-β) or tau, whereas only GFAP and plectin were also significantly enriched (although to a lesser extent) in total insoluble aggregates (Ayyadevara et al., 2016b; Figures 7B–E).

In order to better understand, at the molecular level, binding of PNR502 to target proteins inferred from pull-down experiments (Figure 7A), we performed computational modeling and docking of PNR502. Simulated docking of PNR502 to individual proteins (viz., GFAP and tubulin β chain), and to a GFAP-tubulin complex, was unbiased [i.e., each protein was considered in its entirety (Janganati et al., 2018)]. Results indicate that PNR502 could bind at multiple sites in GFAP, with its most stable complexes forming at the interface between GFAP and tubulin β chain (orange areas in Figure 7F). Interacting regions from the docking studies coincide with the major peptides of GFAP and tubulin β chain identified from the PNR502 pulldown (crimson regions in the enlarged panel of Figure 7G).

To assess the functional importance of these putative target proteins to aggregation, we used RNAi to knock down the orthologous proteins in a *C. elegans* model of amyloid β-induced plaque accrual. CL4176 worms were induced to synthesize human Aβ_1–42_ by upshift to 25.5°C, resulting in paralysis of ~75% of worms within 48 h. RNAi targeting five of the top six PNR502-bound proteins conferred significant rescue from paralysis (Figure 7H; *P* < 0.05 or <0.005). The most effective knockdowns targeted *C. elegans* genes encoding tubulin β chain, GFAP, and histone H2B—increasing the unparalyzed fraction by 2.3-, 2.0-, and 1.7-fold, respectively, over mock-treated controls, whereas plectin knockdown was deleterious. These data implicate GFAP and β tubulin as the principal functional targets of PNR502 but suggest that additional targets may contribute to its remarkable ability to protect against protein aggregation and aggregate-associated phenotypes.

## Discussion

Misfolded and aggregated proteins within cells are degraded by proteasomes and autophagosomes, the principal catabolic agents of protein homeostasis or “proteostasis” (Chen and Yin, 2011; Walther et al., 2015). Our group and others have shown that the protein-aggregate burden increases with age and as a result of age-progressive diseases (David et al., 2010; Ayyadevara et al., 2015, 2016c,d; Walther et al., 2015). Drugs that protect against protein aggregation, or activate pathways for degradation of existing aggregates, may provide effective interventions to prevent or treat neurological disorders such as AD, PD, and HD, as well as other age-associated diseases including hypertension, cancer, and type 2 diabetes (Forloni et al., 2002; Ano Bom et al., 2012; Ayyadevara et al., 2015, 2016b,c,d, 2017; Labbadia and Morimoto, 2015).

The present study focuses on a combretastatin-A4 analog, PNR502, which opposes protein aggregation and rescues many pathological traits associated with induced or age-dependent amyloidosis. Naturally-occurring products have yielded many valuable drugs, such as resveratrol, curcumin, EGCG, small-molecule SIRT1 activators, and many other compounds currently used to treat age-associated diseases including cancer, diabetes, cardiovascular disease, and neurodegenerative diseases (Hubbard and Sinclair, 2014; Ding et al., 2017). Over the past two decades, the pharmaceutical industry has largely relied on high-throughput screening of large, unselected libraries of synthetic chemicals for the discovery of new drugs. The relatively low success rate of this approach has spurred a recent revival of interest in exploring natural products and their analogs, for the discovery of novel drugs (Harvey et al., 2015; Shen, 2015). Natural products continue to play critical roles in drug discovery; for example, 28% of drugs approved between 1981 and 2010 were derived from natural products (Newman and Cragg, 2012). PNR502 does not, to our knowledge, occur naturally; it was synthesized by modification of combretastatin A4 using standard methods of medicinal chemistry (Madadi et al., 2015).

PNR502 was remarkably effective in rescuing *C. elegans* strains that express human Aβ_1–42_ in either muscle or neurons, with the compromise of motility and chemotaxis as their respective endpoints indicating amyloid toxicity. These functional deficits were 40–75% reversed by PNR502 treatment. PNR502 also extended the mean and maximal lifespan of wild-type worms, suggesting that protein aggregation may be a common causal agent limiting lifespan, perhaps by promoting a variety of age-associated disorders.

Cerebral protein aggregation was also significantly reduced in 1-year-old BRI-Aβ_1–42_ mice injected for 10 days with PNR502. Because amyloid aggregates develop over a period of ~12 months in these AD-model mice, the rapid reduction in insoluble aggregates is difficult to reconcile with any mechanism that does not entail clearance of extant amyloid plaque. This surprising inference was reinforced by studies in which *C. elegans* adults that accumulate neuronal amyloid with age, due to leaky expression of a human Aβ transgene, were rescued from impaired chemotaxis by PNR502 treatment for 1–6 days (Figure 2). By day 7, chemotaxis had declined in untreated worms to half of the young-adult level. PNR502 treatment on just the last day was at least as protective as 3 or 6 days of exposure, consistent with either reversal of previously formed aggregates, or prevention of their toxicity. The former interpretation is supported by studies in which PNR502 treatment elicited a similarly rapid reversal of aggregate accrual in a *C. elegans* strain that accumulates fluorescent Q40::YFP aggregates progressively with age.

Human neuroblastoma cells (SY5Y-APP_Sw_), expressing an aggregation- and AD-prone double mutant of APP, accumulate amyloid as measured by fluorescence of amyloid-bound Thioflavin T. Treatment with 5-μM PNR502 for 2 days reduced their amyloid content 50%. Nontransgenic glial and neuronal cells also formed less insoluble aggregate after PNR502 treatment *in vitro*. Exposure to soluble APPα, a pro-inflammatory stimulus, increased glial-cell insoluble aggregates by >60%; this response was fully reversed by treatment with PNR502. Similarly, exposure of NT2 (NTera-2) “preneuronal” carcinoma cells (Pleasure and Lee, 1993) to the inflammatory cytokine IL-1β elicited upregulation of ApoE and of p38/MAPK innate-immune signaling—responses that were fully blocked by PNR502.

We identified candidate target proteins, isolated from AD hippocampus by PNR502-affinity pulldown. The protein with the highest “specific affinity” for PNR502 was GFAP, followed by histone H2B, MBP, and tubulin β chains. Computational modeling and docking indicate that PNR502 binds at the interface between GFAP and tubulin β chain. Drug-adherent peptides in GFAP and tubulin together form a PNR502-binding pocket at the site of their strongest protein-protein interaction, a result also supported by unbiased docking simulations without input from peptide binding (manuscript in preparation). Other identified proteins may have been recovered through weaker affinity for PNR502, or *via* *in vivo* adhesion of GFAP and/or tubulin to other proteins in AD aggregates. The results should be interpreted cautiously, since the most abundant protein targets may not be the most biologically relevant proteins, i.e., those responsible for reduced aggregation.

GFAP is an intermediate-filament protein that is not only most conspicuous in astrocytes but also expressed in many other cell types. Like tubulin and actin, GFAP is critical for cytoskeletal integrity, with additional roles in cell signaling, morphology, and focal adhesions (Moeton et al., 2016). GFAP also provides structural support to neurons and regulates neurotransmitter levels (Yang and Wang, 2015). GFAP biosynthesis is induced in activated glial cells, and its presence in cerebrospinal fluid is a biomarker of AD or related dementias (Ishiki et al., 2016). Knockout mice completely lacking GFAP are hypersensitive to traumatic brain injury (Nawashiro et al., 1998).

HIV-1-associated dementia is mediated by the HIV tat protein (*trans* activator of transcription; Irish et al., 2009). Tat-mediated GFAP aggregation elicits an unfolded protein response in astrocytes where it causes ER (endoplasmic reticulum) stress leading to neurotoxicity (Fan and He, 2016). Alexandria disease, which impacts the central nervous system, can arise from an aggregation-prone GFAP mutation (Lee et al., 2017). We previously showed that GFAP is enriched in hippocampal aggregates from AD, relative to controls (Ayyadevara et al., 2016b), suggesting that GFAP sequestration into aggregates might contribute to AD pathology.

## Conclusion

PNR502, a novel drug, opposes protein aggregation in a wide variety of AD-model systems, including 4 human cell types, and both *C. elegans* and mouse models of Aβ_1–42_ amyloidopathy. In mouse cerebra and in nematodes, it appears to reverse aggregation that had occurred previously. Further studies of this compound, and of subsequent-generation drugs that target GFAP, tubulin, and their interface, might provide even more effective therapeutic agents for the prevention or reversal of neurological disorders such as AD.

## Data Availability Statement

The raw data supporting the conclusions of this article will be made available by the authors, without undue reservation, to any qualified researcher. Proteomics data, for proteins identified by pulldown with biotinyl-PNR502, are available at https://data.mendeley.com/datasets/pvyz6r8kgn/4.

## Author Contributions

NP, SBB, and PC were responsible for the concept and synthesis of PNR502 and other drug libraries mentioned herein. SA, MB, SWB, WG, and RS designed and interpreted the experiments. SK, SA, MB, AG, LL, and RA performed the experiments. The manuscript was written by SA, MB, and RS with additional contributions from NP and SWB.

## Conflict of Interest

The authors declare that the research was conducted in the absence of any commercial or financial relationships that could be construed as a potential conflict of interest. The University of Arkansas for Medical Sciences (UAMS) and the U.S. Government (through the Dept. of Veteran Affairs) jointly hold a patent on the compound PNR502 for protection against Alzheimer’s and other neurodegenerative diseases (PCT filing WO 2018/144910 Al). A potential royalty stream to RS, PC and SA may occur consistent with the policies of these institutions.

## Footnotes

^1^http://www.rcsb.org

^2^https://zhanglab.ccmb.med.umich.edu/I-TASSER

^3^http://hex.loria.fr/

^4^vina.scripps.edu
